# The use of serology for trachoma surveillance: Current status and priorities for future investigation

**DOI:** 10.1371/journal.pntd.0008316

**Published:** 2020-09-24

**Authors:** Diana L. Martin, Martha Idalí Saboyà-Díaz, Aida Abashawl, Wondu Alemayeh, Sarah Gwyn, Pamela J. Hooper, Jeremy Keenan, Khumbo Kalua, Celia Landmann Szwarcwald, Scott Nash, Catherine Oldenburg, Sheila K. West, Michael White, Anthony W. Solomon

**Affiliations:** 1 Division of Parasitic Diseases and Malaria, Centers for Disease Control and Prevention, Atlanta, Georgia, United States of America; 2 Communicable Diseases and Environmental Determinants of Health Department, Pan-American Health Organization, Washington, DC, United States of America; 3 Berhan Public Health and Eye Care Consultancy, Addis Ababa, Ethiopia; 4 The Fred Hollows Foundation, Addis Ababa, Ethiopia; 5 International Trachoma Initiative, Task Force for Global Health, Decatur, Georgia, United States of America; 6 Francis I. Proctor Foundation, University of California at San Francisco, San Francisco, California, United States of America; 7 Department of Ophthalmology, University of California at San Francisco, San Francisco, California, United States of America; 8 Department of Ophthalmology, University of Malawi, College of Medicine Blantyre, Malawi; 9 Institute of Scientific and Technological Communication and information in Health, Oswaldo Cruz Foundation, Rio de Janeiro, Brazil; 10 Trachoma Control Program, The Carter Center, Atlanta, Georgia, United States of America; 11 Department of Epidemiology and Biostatistics, University of California at San Francisco, San Francisco, California, United States of America; 12 Wilmer Eye Institute, Johns Hopkins School of Medicine, Baltimore, Maryland, United States of America; 13 Malaria: Parasites & Hosts, Department of Parasites and Insect Vectors, Institut Pasteur, Paris, France; 14 Department of Control of Neglected Tropical Diseases, World Health Organization, Geneva, Switzerland; RTI International, UNITED REPUBLIC OF TANZANIA

## Background

Programs seeking to eliminate the eye disease trachoma use prevalence of the clinical sign trachomatous inflammation–follicular (TF) in 1- to 9-year-olds as a proxy for population-level transmission of ocular *Chlamydia trachomatis* (*Ct*). TF prevalence determines the need for the A, F, and E components of the “SAFE” (surgery, antibiotics, facial cleanliness, environmental improvement) strategy. Ocular *Ct* infection, like its associated signs of conjunctival inflammation, is most common and most intense in young children [1, 2] who are repeatedly infected in areas of active transmission [3]: a model suggests that people can be infected more than 150 times in their lifetime [4]. Repeated infection leads to multiple episodes of TF plus more intense conjunctival inflammation and eventually conjunctival scarring (trachomatous scarring [TS])[5, 6]. Contraction of conjunctival scar can, over a period of years or decades, cause the upper eyelid to turn in and the eyelashes to rub against the eyeball (trachomatous trichiasis [TT]), which can lead to corneal opacity (CO) and blindness.

The World Health Organization (WHO) set trachoma’s elimination prevalence thresholds as <5% TF in 1- to 9-year-olds, and <0.2% TT unknown to the health system in ≥15-year-olds, in evaluation units (EUs) of 100,000–250,000 people [7]. Once these criteria are met in all previously endemic areas and provisions are in place for identification and management of incident cases of TT, a country may apply to WHO for validation of elimination of trachoma as a public health problem. However, no guidance is in place for how programs should monitor for potential recrudescence (in the form of increased *Ct* transmission or its corollary, increased TF prevalence) after the elimination criteria for TF have been met.

A postvalidation surveillance system for trachoma that could provide a quantitative measure of ocular *Ct* transmission would be valuable. Serological testing has a potential role in this, in the same way that antibody acquisition is used as a proxy measure of transmission for malaria and several other infectious diseases [8, 9]. An assumption underlying the potential use of serological surveillance for trachoma is that an antibody response becomes detectable in an individual’s blood only after multiple previous exposures to infection. This is the most parsimonious explanation for the observation that in trachoma-endemic populations, ocular *Ct* infection is seen in children aged <1 year, but age-specific antibody prevalence only starts to exceed infection prevalence at the age of 3–4 years [10]. In contrast, in a nonhuman primate model of trachoma, single exposures have been observed to induce detectable serum antibodies for at least 15 weeks [11].

The concept of repeated exposure may be important here, just as it is in the development of the blinding complications of trachoma. The pathological state initiated by TS only begins after multiple infections [4]. Therefore, one or two childhood infections with ocular *Ct* strains, or ocular exposure to genital *Ct* strains at the time of delivery or subsequently, should not confer a risk of subsequent TT. A measure of repeated exposure would be beneficial in identifying trends in population-level transmission of public health significance. We note, however, that even if multiple exposures are not necessary to generate a detectable antibody response, anti-*Ct* antibodies may still have programmatic value because they provide information about *Ct* transmission at community level, as we detail in this paper.

From October 9 to 10, 2018, a technical consultation was convened at the Task Force for Global Health in Decatur, Georgia, United States, to review available data on serological surveillance for trachoma, discuss ongoing studies, and identify knowledge gaps to plan future work. Participants included disease experts, laboratory and field scientists, laboratory test developers, academic researchers, control program managers, and mathematical modelers from four continents. This article summarizes the outcomes of this meeting and lays out research priorities to fully evaluate whether and how serology could be used for postvalidation surveillance by trachoma programs.

## What we need to know

At present, there are three questions being considered in parallel in studies evaluating the use of serological surveillance for trachoma:

1. Can—and should—antibody testing be used for trachoma surveillance?
2. What assays should be used to measure the presence, absence, or intensity of anti-*Ct* antibodies in an individual?
3. How might surveillance using anti-*Ct* antibodies be deployed at the population level?

## Progress to date

### Rationale for serological surveillance of trachoma and pilot community-level studies

Inspired by a considerable body of research by earlier investigators [12, 13], antibody-based testing for trachoma surveillance was proposed at a 2010 consultation on diagnostic tools for neglected tropical disease (NTD) programs [14]. Following this meeting, a series of community-level studies were conducted that demonstrated a general correlation between TF prevalence and antibody seroprevalence in 1- to 9-year-olds [10, 15, 16]. Where trachoma was endemic prior to mass drug administration (MDA), the antibody seroprevalence was typically 2–3 times that of TF prevalence, likely representing greater longevity of antibody-secreting plasma cells than the follicles that characterize TF. Additionally, an increase in antibody seroprevalence with increasing age was apparent in trachoma-endemic communities [10, 15]. This age-dependent increase in the proportion of 1- to 9-year-olds seropositive to antibodies against *Ct* antigens likely reflects cumulative exposure to ocular *Ct* in childhood. In communities that had eliminated trachoma, antibody positivity rates were low, with little to no age-related increase [17].

### Population-level studies

All published and known unpublished data from population-level studies that included serological data collection were presented at the meeting. All data were collected under protocols approved by the appropriate Institutional Review Boards from the country and affiliated institutions. This included surveys from 38 EUs in 13 countries at all programmatic stages at which surveys are generally implemented (baseline, impact survey, prevalidation surveillance survey).

At baseline, there are currently few endemic EUs that have yet to be mapped or receive treatment, so opportunities to obtain preintervention serological data are somewhat scarce. Of note, high TF prevalence (28%) was accompanied by high antibody prevalence (53%) and a sharp increase in antibody prevalence with age in Kiribati [18]. This is not always seen in Pacific Island nations, as described below.

Seroprevalence in younger children is thought to reflect current and recent *Ct* transmission intensity. It is expected to be low at the time of impact surveys, which have been undertaken after 3–10 annual rounds of antibiotic MDA. What has been observed to date is that at impact survey, antibody prevalence remains high in settings where TF remains above 5%. For example, in 2 EUs evaluated in the Amhara region in Ethiopia, TF prevalence has been slow to decline despite 8–10 rounds of MDA, and antibody levels remain high, with steep increases in seroprevalence rates among 1- to 9-year-olds, implying continuing high-intensity transmission. Similarly, in Niger, a TF prevalence of 7.5% after three rounds of MDA corresponded to an antibody prevalence of 29% in 1- to 5-year-olds [19]. By contrast, where TF prevalence has been below the elimination threshold at impact survey, antibody prevalence in 1- to 9-year-olds has typically also been low. Examples of this scenario have been documented in The Gambia [20] and Uganda [21].

Similarly, when serology has been included as part of EU-level prevalidation surveillance surveys, antibody prevalence has been found to be low (<7.5% and often <2%), with no or barely detectable increases with increasing age [22–24].

### Assays for measuring antibody responses

Important considerations in interpreting absolute seroprevalence are the assay used to measure antibody responses and the method used to generate cutoffs. Initial studies looking at community-wide seroprevalence used a multiplex bead-based assay (MBA) [10, 15, 17]. As interest in evaluating seroprevalence increased, especially among laboratories located in trachoma-endemic countries where access to MBA tends to be more limited, the test was adapted to ELISA. We note that the methodologies and performance characteristics of other ELISAs to detect antibodies to the *Ct* antigen plasmid gene product 3 (Pgp3) have previously been published [25–28]; a recent refinement was the inclusion of a normalization standard for assay quality control when working in a variety of laboratory settings [29]. Testing for anti-Pgp3 antibodies was also transferred to a lateral flow assay (LFA) format, which had similar performance characteristics to the MBA at individual [29, 30] and population [31] levels. Feedback from laboratories and field staff was taken into consideration and used to revise and optimize the tests for programmatic use. For example, the 30-minute development time for the LFA results in a distinct test line and high sensitivity compared with MBA, but this development time slowed down field teams going from house to house. For population-level surveillance, an inexpensive, easy-to-use test is an asset, whereas a point-of-care test is not needed because a positive test is not an indication for treatment of the individual. Rather than producing a test with a shorter development time, the usage scenario was taken into account, and the LFA was positioned for laboratory deployment [32].

### Using serology to understand transmission dynamics

Much of the existing data support the conclusion that increases in seroprevalence with increasing age do, as hypothesized, reflect cumulative exposure of individuals to *Ct*. A recent study analyzed serological data from nine populations using various modeling approaches to estimate population-level seroconversion and seroreversion rates [33]. Pre- and post-MDA data from three communities in Nepal illustrate the use of population-level seroconversion estimates as a measure of changes in *Ct* transmission intensity. Prior to MDA, when trachoma was still endemic in these communities in Nepal, the average time to seroconversion of an individual was estimated as 8 years. At 4 years after MDA, the average time to seroconversion was estimated as 125 years. The population’s seroreversion half-life—the time taken for 50% of a population of seropositive individuals to revert to being seronegative—was 26 years.

The mechanics of cumulative exposure, an increase in the number of individuals that have ever been exposed with increasing age, are worth considering. In the case of chronic infections, such as human herpesviruses, as people become and remain infected, they also become and remain seropositive. With age, the proportions of individuals infected and seropositive generally increase at the same rate. In the case of organisms associated with acute or subacute infection and possible repeated exposure, such as ocular *Ct*, as people become infected, they may become seropositive. After they clear infection, some proportion of seropositive individuals become seronegative. Individuals who subsequently become reinfected may become seropositive again. As people acquire a greater number of lifetime exposures, it is probable that a smaller and smaller proportion will become seronegative following infection clearance because of the development of long-lived antibody-secreting plasma cells. Therefore, if the incidence of *Ct* infection is relatively constant across different ages, the prevalence of seropositivity would increase with age.

The exact dynamics of ocular *Ct* infection are largely unknown. For example, it is unclear how frequently children are infected in settings with various levels of TF prevalence [34], how soon after infection children become antibody positive, whether it takes multiple infections (and/or particular bacterial loads) for a peripheral antibody response to become patent or to develop long-lived plasma cells, and how frequently seroreversion occurs after varying numbers of infections, among other questions. Generating robust answers to these questions through observational studies alone would be formidably difficult. We can, however, use empiric data to develop models that may allow the potential programmatic utility of serology to be further explored.

Longitudinal data on seroconversion and seroreversion events collected at the individual level could provide important biological insights and critical data for model development, as has been done in one study [35]. Cross-sectional data provide a snapshot of antibody patterns in a population and information on historical exposure to the pathogen. Collecting multiple cross sections in a population at different stages of elimination can provide estimations of the seroconversion and seroreversion rates.

### Selecting optimal cutoffs for seropositivity

Different approaches for determining the cutoff for seropositivity continue to be explored [21], and as already noted, different studies have employed different assay formats. Alternate methods are likely to lead to divergent overall seroprevalence estimates. But focusing on the rate at which the population seroconverts may overcome vagaries in absolute seroprevalence estimates attributable in part to the use of different tests or cutoff determinations. For example, using samples from prevalidation surveys in Ghana, antibody responses were measured in different laboratories by ELISA using a cutoff determined by a fixed mixture model and by MBA using a receiver operating characteristic (ROC) curve cutoff. The overall seroprevalence estimates were different (even if not dramatically different): 5.5% (95% CI 4.8–6.3) by ELISA and 4.3% (95% CI 3.7–4.9) using the MBA. But the seroconversion rates were essentially identical: 1.3 yearly seroconversion events per 100 children by ELISA and 1.2 by MBA. Therefore, although it is impossible to know whether 5.5 or 4.3 was the “true” seroprevalence, the SCR data suggest that it may not matter, as neither assay suggested significant population-level seroconversion, implying that transmission intensity was likely too low to have public health importance.

### Settings where indicators diverge

As data from a growing number of population-level surveys that have included serological testing become available, we are identifying more sites that deviate from expectations. Some of these are surveys in which data on antibody and ocular *Ct* infection were specifically investigated as alternative indicators to TF in populations in which there was high TF prevalence in children but little or no TT in adults. The divergence between seroprevalence and prevalence of TF in some of these settings may reflect an underlying epidemiology where the TF phenotype was not actually due to trachoma.

For example, the epidemiology of trachoma, ocular *Ct* infection, and anti-*Ct* antibodies in the Melanesian Pacific Islands has been examined in some detail. Data reviewed at the meeting from multiple EUs of the Solomon Islands, Vanuatu, and Papua New Guinea suggest that moderately high levels of TF in these populations are not reflective of ongoing intense ocular *Ct* transmission: the prevalence of ocular *Ct* infection is very low, and there is no increase in seropositivity among 1- to 9-year-olds. However, in the Solomon Islands, the absolute prevalence of antibody positivity in 1- to 9-year-olds is higher than that seen in other areas with low-intensity ocular *Ct* transmission [36]. The Solomon Islands have a high prevalence of urogenital *Ct* infection in women of reproductive age [37], which might explain this finding, potentially related to exposure of newborns to urogenital strains at the time of delivery, or exposure of children to ocular infection with urogenital strains through poor parental hand hygiene.

Data in which the relationship between infection and disease diverge significantly from the expected paradigm have also been encountered in West Hararge Zone, Oromia, Ethiopia; these data are currently being more fully explored.

### Ensuring high-quality data

Processes to ensure data quality and consistency are critical for any potential surveillance tool [38]. Assays used to measure antibody responses need to be appropriately validated. The relative performance of each of the currently available platforms (MBA, ELISA, and LFA) was reviewed at the meeting, with a focus on the LFA, which is likely to be the least expensive option without sacrificing throughput.

An external reference standard would further strengthen assay validation and allow better test standardization across laboratories. Progress toward the development of a chimeric antibody that could be used in test development and as a reference standard for plate controls and antibody quantitation was presented at the meeting. The development process starts by generating a panel of mouse monoclonal antibodies to screen for reactivity to Pgp3; this foundational step has already been accomplished. The Pgp3-binding paratope for multiple antibody-secreting clones has been sequenced and is in the process of being cloned into the backbone of the human IgG constant region.

Alongside assay-level validation, work is required to confirm the acceptability of sample collection for trachoma surveillance among endemic populations and the availability of appropriate cadres of staff to reliably collect and process blood samples.

### Meeting outcomes and research priorities

The overriding question for the technical consultation was as follows: Is there now sufficient evidence for the global trachoma community to consider deploying serology for postvalidation disease surveillance?

Based on the discussion of the evidence available at the meeting, the answer to this question is not yet. However, meeting participants agreed further investigation was warranted and defined a set of priorities for future studies. These priorities are listed below.

### Collection of data in particular epidemiological settings should be prioritized

We identified that data collection from baseline, preintervention settings are of primary importance. There are currently few serological data from antibiotic-MDA–naïve EUs with moderate to high (>20%) TF prevalence in 1- to 9-year-olds. These are the epidemiological settings in which it would be expected to see sharp increases in seroprevalence with age in 1- to 9-year-olds, but further data are needed to adequately document this. Many EU-level studies undertaken to date have been carried out in settings (such as the Pacific Island countries) in which TF is suspected to overestimate trachoma’s likely public health impact, and more data representative of unquestionably trachoma-endemic settings are needed to more firmly establish points of reference. A limitation to obtaining baseline data from populations with moderate to high trachoma prevalence is the considerable scale-up of mapping and interventions against trachoma since 2012 [39]. Opportunities, however, are being taken as they arise.

Although data from baseline high-prevalence environments are highly desirable, studies in all programmatic settings will continue to provide valuable information for evaluation of serology for trachoma surveillance.

To better estimate seroconversion rates and to identify individual seroconversion events, which are critical parameters for modeling how best to use serology as a programmatic tool, settings where individuals and populations can be followed longitudinally in areas of varying trachoma prevalence have been identified and started in one formerly hyperendemic site [35]. Longitudinal cohort studies with monthly sampling of large populations over 1 year have been scheduled for implementation in Senegal, Ethiopia, and Papua New Guinea between 2020 and 2022. An important consideration when undertaking and interpreting individual-level studies is recognizing that TF is a relatively blunt tool for determining the presence or absence of *Ct*-induced inflammation; in particular, when using the WHO simplified grading system [40], lesser degrees of inflammation than that required to meet the definition of TF are ignored [41].

Trachoma is not the only cause of the TF phenotype, and the etiology of follicular conjunctival inflammation may vary by region. The absence of significant levels of *Ct* infection or of a variety of host responses to *Ct* infection, including antibody, raises questions about the appropriateness of interventions against active trachoma in certain settings [36]. The possibility that a high prevalence of TF may not reflect high levels of ocular *Ct* transmission in geographical locations other than Melanesia should be investigated.

### Populations likely to be at high risk of recrudescence should be prioritized for serosurveillance

A foundation for any consideration of postvalidation trachoma surveillance is a hypothesis for what recrudescence would actually look like. The expert opinion at the consultation was that recrudescence would include ocular *Ct* infection in children younger than 5 years old, and it might be predicted that surveillance would ultimately focus on children within this range. Therefore, it is desirable that children aged 1–5 years be included in all studies evaluating the use of serology for trachoma surveillance. Additionally, work should be undertaken to identify specific districts predicted to be at greatest risk for recrudescence to be invited to participate in serological studies. This may include districts that took more than the recommended minimum number of rounds of MDA to achieve TF <5%.

### The potential contribution of antibody signals derived from urogenital rather than ocular *Ct* strains is a concern, and studies to understand (and if possible, subtract) this signal should be pursued

Most EUs in which postvalidation surveillance for trachoma will occur do not have robust surveillance for urogenital *Ct*, so any use of serology for surveillance will have to contend with occasional neonatal and childhood exposure to urogenital *Ct* strains, without knowledge of local prevalence of sexually transmitted infections (STIs). Further work to delineate the contribution of urogenital *Ct* strain infection to overall seroprevalence will be critical to properly understanding how serology could be used for trachoma surveillance. Collection of serological data from settings with robust STI surveillance would allow us to predict seroprevalence in young children in the near absence of exposure to urogenital *Ct* strains and to better determine the longevity of the antibody response. High seroprevalence in older children in such settings will most likely reflect long-lived antibody responses due to previous exposure to ocular *Ct* strains, rather than recent ocular exposure to urogenital *Ct*.

Although the contribution of an STI-derived antibody signal is of concern, it may be a surmountable problem. In addition to looking at the profile of the seroprevalence curve within the 1- to 9-year-old age group and taking into account data on prevalence of urogenital infection in adults (where available), there may be measures of recent infection that would be valuable in distinguishing intermittent exposure to urogenital *Ct* from ongoing transmission of ocular *Ct*. Among the avenues that could be pursued for this is examination of isotype-specific antibody responses. Antibodies of the IgG3 isotype have been shown to decline following treatment of urogenital *Ct* infection, whereas IgG1 responses remain high [42]. There may also be specific antigens that generate short-lived antibody responses. It may also be possible to identify antigens that are specific for ocular strains of *Ct*.

### Studies to evaluate serological surveillance for trachoma should be undertaken with the highest level of epidemiological and laboratory rigor

Survey designs for collection of dried blood spots should be epidemiologically rigorous, conforming to WHO recommendations for population-based trachoma prevalence surveys [43]. This includes collection of appropriate demographic data on participants. To correlate population-level seroprevalence data with TF and infection data, it is recommended that, wherever possible, collection of data on TF and ocular *Ct* infection should be included in studies in which antibody data will be generated.

Antibody testing should include testing for antibodies to Pgp3 antigen, at a minimum, because most datasets generated to date include data on anti-Pgp3. To increase the number and availability of samples to validate improved tests for anti-*Ct* antibodies and to carry out additional studies to improve antibody testing for ocular *Ct* for surveillance, broad informed consent for future use of the samples should be obtained from persons participating in trachoma studies [44].

A set of standard operating procedures for laboratory analyses of dried blood spots to detect antibodies against *Ct* should be developed and circulated to participating laboratories. For example, process documents for identifying critical reagents for large-scale serosurveys to ensure that reagent lots are not changed midanalysis have been developed. The highest levels of quality control for antibody testing can be achieved with external standards. To this end, a positive control chimeric antibody is under development, as noted above.

## Conclusions

To date, nine countries have validated elimination of trachoma as a public health problem: Cambodia, China, Ghana, Islamic Republic of Iran, Lao People’s Democratic Republic, Mexico, Morocco, Nepal, and Oman. Others are soon to follow, and guidance for postvalidation surveillance to detect recrudescence if and when it occurs will be critical to maintaining hard-won programmatic gains. Serologic surveillance has potential for providing a measure of ocular *Ct* transmission to assess changes in transmission over time. Strategies and tools for postvalidation surveillance should be based on high-quality evidence. We provide here an assessment of the state of research of serological tools for trachoma surveillance and outline priorities and best practices for obtaining further useful data.

## Ethics statement

All studies were conducted with approval from appropriate local and national ethics committees. Informed consent or parental consent was obtained from all participants.
